# Ketamine Alters Functional Plasticity of Astroglia: An Implication for Antidepressant Effect

**DOI:** 10.3390/life11060573

**Published:** 2021-06-17

**Authors:** Matjaž Stenovec

**Affiliations:** 1 Celica BIOMEDICAL, Tehnološki Park 24, 1000 Ljubljana, Slovenia; matjaz.stenovec@mf.uni-lj.si; Tel.: +386-1-543-7081; 2 Laboratory of Neuroendocrinology-Molecular Cell Physiology, Faculty of Medicine, Institute of Pathophysiology, University of Ljubljana, Zaloška 4, 1000 Ljubljana, Slovenia

**Keywords:** astrocytes, ketamine, cAMP, exocytosis, endocytosis, fusion pore, K_ir_4.1, vesicle mobility, cholesterol

## Abstract

Ketamine, a non-competitive *N*–methyl–d–aspartate receptor (NMDAR) antagonist, exerts a rapid, potent and long-lasting antidepressant effect, although the cellular and molecular mechanisms of this action are yet to be clarified. In addition to targeting neuronal NMDARs fundamental for synaptic transmission, ketamine also affects the function of astrocytes, the key homeostatic cells of the central nervous system that contribute to pathophysiology of major depressive disorder. Here, I review studies revealing that (sub)anesthetic doses of ketamine elevate intracellular cAMP concentration ([cAMP]_i_) in astrocytes, attenuate stimulus-evoked astrocyte calcium signaling, which regulates exocytotic secretion of gliosignaling molecules, and stabilize the vesicle fusion pore in a narrow configuration, possibly hindering cargo discharge or vesicle recycling. Next, I discuss how ketamine affects astrocyte capacity to control extracellular K^+^ by reducing vesicular delivery of the inward rectifying potassium channel (K_ir_4.1) to the plasmalemma that reduces the surface density of Kir4.1. Modified astroglial K^+^ buffering impacts upon neuronal firing pattern as demonstrated in lateral habenula in a rat model of depression. Finally, I highlight the discovery that ketamine rapidly redistributes cholesterol in the astrocyte plasmalemma, which may alter the flux of cholesterol to neurons. This structural modification may further modulate a host of processes that synergistically contribute to ketamine’s rapid antidepressant action.

## 1. Astrocytes, Molecularly Heterogeneous Keepers of the Central Nervous System

Astrocytes are morphologically and functionally heterogeneous glial cells that principally provide for the homeostasis and defense of the central nervous system (CNS) [1,2]. In the human brain, astrocytes account for 20–40% (region dependent) of the glial cell population [1,3,4,5,6,7,8,9]. On the basis of their anatomic location, astrocytes were initially classified into protoplasmic and fibrous astrocytes located in the grey and white matter, respectively [1]. Subsequent classifications were made on the basis of astrocyte morphology and the expression of various protein markers, such as glial fibrillary acidic protein (GFAP, intermediate filament protein that is expressed in a subpopulation of astrocytes with considerable region variability, and is upregulated in reactive astrocytes; [10,11]) and vimentin (intermediate filament protein that is expressed in immature astrocytes, in subpopulations of protoplasmic and fibrous astrocytes, in Bergmann glia, and is upregulated in reactive astrocytes; [12,13,14]), S100B protein (Ca^2+^-binding protein, which act as Ca^2+^ buffer and possibly as Ca^2+^ sensor; [15,16]), aldehyde dehydrogenase 1 (ALDH1L1, an enzyme involved in folate metabolism that contributes to nucleotide biosynthesis and cell division; [17,18]), glutamine synthetase (cytosolic enzyme converting ammonia and glutamate into glutamine; [19,20,21,22]), glutamate transporters EAAT–1 (GLAST, predominantly expressed in cerebellum; [23,24,25]) and EAAT–2 (GLT–1, the main glutamate transporter in other brain regions; [26]), aquaporin 4 (AQP4, preferentially localized to the endfeet of healthy astrocytes; [27,28]), and hemichannels connexins (Cxs), such as Cx43 and Cx30, localized to the astrocyte plasmallema and concentrated in the endfeet [29,30].

Astrocytes in vitro almost invariably express GFAP, whereas in vivo, subpopulations of GFAP-positive astrocytes vary in size. In cerebellum, virtually all Bergmann glia are GFAP immuno-positive [31], whereas in the juvenile hippocampus, ~80% or more astrocytes are GFAP immuno-positive [16,32]. In contrast, in the adult rat hippocampus, numerous astrocytes are not stained with anti-GFAP antibodies [33], whereas in the cerebral cortex of mature rodents, only 20% of protoplasmic astrocytes are GFAP immuno-positive [34]. In CNS, astrocytes are distributed in a region-specific manner; they are much sparser in areas clustered with neuronal somatic synapses and blood vessels than in areas with dendrites and axons, where thin processes of astrocytes may contact blood vessels and nodes of Ranvier [6]. By the means of multiple fine processes, astrocytes enwrap synapses and express receptors for neurotransmitters and neuromodulators [35]. In response to neuronal firing, astrocytes increase free intracellular calcium concentration ([Ca^2+^]_i_) and may subsequently release gliotransmitters that regulate synaptic transmission and plasticity on the timescale of tens of milliseconds to minutes [36,37]. To emphasize the close relationship between neurons and astrocytes at glutamatergic synapses, the term “tripartite synapse” was coined in the past [38]. This concept has now been broadened to other synapses, including the monoaminergic synapses to indicate that astrocytes modulate information processing in the neuronal networks [39]. Astrocytes not only release gliotransmitters that act fast inside synapses, but also release factors that act slowly, on a timescale of minutes to days, and affect extra-synaptic targets that regulate energy supply, metabolism, development and inflammation. These molecules include metabolic substrates, growth/trophic factors, and inflammatory mediators [39,40]. Astrocytes interconnected via gap junctions, which allow direct passage of nucleotides (ATP and ADP), second messengers, metabolites with small molecular weight and ions [41], form an astrocyte syncytium [42]. In rodents, and possibly in other mammals as well, protoplasmic astrocytes occupy spatially non-overlapping territorial domains that encompass vasculature and neurons [32]. In humans and non-human primates, astrocytes with very long interlaminar processes may extend beyond these territories [43]. In the human brain, an astrocyte can physically contact up to ~2 million synapses, whereas in the mouse brain it contacts fewer; up to ~0.1 million synapses [35,44]. The perivascular astrocytes with highly ramified processes envelop most of the capillary surface via endfeet [45] that mediate neurovascular coupling and maintain the integrity of the blood–brain barrier [46].

During development, astrocytes in the form of radial glia guide migrating neurons towards neocortical destinations and instruct them to form synapses [47]. Throughout life, astrocytes promote the survival of neurons [48] and signal back to them by the exocytotic release of gliosignaling molecules [40,49] and factors that regulate connectivity and synaptic strength, which are pivotal in learning and memory formation [50,51]. Following CNS insults, astrocytes undergo evolutionary conserved program of reactive astrogliosis (reviewed in the consensus paper by Escartin and colleagues [52]) that drives changes in gene expression, as well as morphological, biochemical, metabolic, and physiological remodeling, which ultimately result in gain of new function(s) or loss or even upregulation of homeostatic ones. Astrocyte reactivation is thus not a single all-or-none response, but instead, a diverse continuum of graded and context-specific responses that may result in adaptive or maladaptive effects [6,53]. As astrocytes affect nearly every aspect of neuronal excitability and function (they are essential for mechanical support, energy supply and the delivery of metabolites to neurons, for ion, water and neurotransmitter homeostasis, neurogenesis, gliogenesis and neurite outgrowth, synapse development, function and plasticity, glucose sensing and transport, energy storage, blood flow regulation, sleep and circadian rhythm modulation, memory processing and storage, and sensory processing, motor coordination, emotion and cognition; all reviewed in [1]), they also play a role in pathogenesis of neurodegenerative disorders including Alzheimer’s disease (AD), Huntington disease (HD), Parkinson disease, amyotrophic lateral sclerosis (ALS), and neuropsychiatric disorders. Indeed, rapidly mounting evidence indicates that astrocytic malfunction underlies many, if not all, neurological, neuropsychiatric and neurodegenerative disorders [2,54]. Astrocytes therefore represent an alternative therapeutic cell target, and many drugs we currently use in neurology may exert their beneficial effects through astrocytes [55]. It is thus possible that conventional antidepressants achieve the therapeutic effect by not only affecting neuronal, but also astrocyte plasticity, by altering the morphology, number and function of astrocytes that affect synaptogenesis, synaptic strength and stability [56].

## 2. Astrocytes in Major Depressive Disorder

As astrocytes stabilize the neuronal environment, provide metabolites and growth factors to neurons, and play a role in signal integration and brain information processing [7,36,57,58], pathologic alteration of astrocytes could destabilize operation of neural circuits implicated in mood regulation. Unsurprisingly, diverse studies have revealed a link between astrocyte impairment and psychiatric disorders such as schizophrenia, bipolar disorder or major depressive disorder (MDD) [59,60,61,62]. However, unlike in neurotrauma and in neurodegenerative diseases, astrocyte atrophy and degeneration without signs of reactivity are characteristic in psychiatric disorders, in particular in MDD [61,63]. In post-mortem samples from depressed individuals, a reduced packing density or number of Nissl-stained astrocyte populations [64,65,66,67,68,69] was found in various brain regions including the anterior cingulate cortex [66,67], subgenual cortex [68], dorsolateral prefrontal cortex [65,69], orbitofrontal cortex [69], and amygdala [64]. In the white matter of post-mortem human brain tissue and in rats subjected to chronic stress, the densities of astrocytes and glial fibrillary acidic protein (GFAP)-positive area fraction were substantially reduced when compared with healthy controls [61]. In rodents exhibiting depressive behavior, reduced GFAP expression and density of astrocytes was also found in the grey matter [70,71]. In animal models of attention deficit disorder and MDD, the downregulation of classical astrocyte markers such as astrocyte-specific connexins, plasmalemmal glutamate transporters, aquaporin 4, and glutamine synthetase was reported [72,73,74]. Pharmacological inhibition of astroglial glutamate transporters [75] and gap junction connectivity [76] triggered anhedonia indicative of depression. The abnormalities in the amount of glutamate at synapses controlled by astrocytes have also been linked to depression, anxiety, and schizophrenia [77]. In addition to glutamate, astrocytes also recycle serotonin, dopamine, and other monoamines implicated in psychiatric disorders. Astrocyte activation via dopamine D2 receptors suppresses neuroinflammation in the CNS [78] that may be the root cause for some forms of major depression [79]. Data gathered from post-mortem studies suggest reduced astrocyte communication with other cell types. In depressed individuals, reduced expression of glutamate transporters indicates reduced capacity for astrocytic uptake of glutamate released by neurons into the synaptic cleft [73,80,81,82,83,84]. Additionally, a decreased release of neurotrophic factors from astrocytes was reported in the locus coeruleus of MDD patients and in rats exposed to chronic social defeat [85]. As molecular and physiological features of astrocytes are not hardwired throughout life but depend on neuronal cues that regulate and determine their functional properties in healthy and injured brains [86,87,88,89], astrocytes are potentially of significance as a drug target for major depressive disorder.

## 3. Antidepressants Affect Astrocyte Plasticity

New awareness of astrocyte importance for normal brain function provided fresh momentum to an old idea of astrocytes as causative elements in the pathogenesis of psychiatric disorders. According to this view, astrocytes are target cells for psychiatric drugs. Consistently, several classic antidepressants (such as Li^+^, valproic acid or fluoxetine) affect astrocyte signaling cascades and modify the expression of various receptors and transporters responsible for CNS homeostasis and for the support of synaptic transmission [56,90,91,92,93]. Astroglial serotonin 5–HT_2B_ receptors interact with serotonin-specific reuptake inhibitors (SSRI) fluoxetine, fluovoxanine, paroxetine, citalopram, and sertraline [94]. Fluoxetine and other SSRI interacting with astroglial 5–HT_2B_ receptors trigger Ca^2+^ signaling [95] and transactivation of epidermal growth factor receptors linked to MAPK/ERK and PI3K/AKT signaling cascades that modulate multiple homeostatic pathways including expression of glutamate transporters, activity of Na^+^–H^+^ exchanger, astroglial secretion and glucose metabolism [91,92,96,97,98]. Intra-hippocampal injection of fluoroacetate metabolically inhibits astrocytes and prevents anti-depressant action of imipramine [99]. The oldest classical antidepressant Li^+^ acting via lysyl oxidase affects morphogenesis and proliferation of astrocytes [93]. GFAP expression, which is generally suppressed in depressive disorders [100,101], is upregulated by the electroconvulsive therapy in patients suffering from MDD [102]. Transcranial direct current stimulation triggers cortex-wide global elevation of cytosolic Ca^2+^ in astrocytes mediated by α1 adrenoreceptor activation and astroglia-specific InsP_3_ receptors type 2 [103].

## 4. Ketamine, the Fastest Antidepressant

Ketamine, (S)–(+) and (R)–(–)–2–(2–chlorophenyl)–2–(methylamino) cyclohexanone, is an arylcycloalkylamine infrequently used as an anesthetic in the human and veterinary medicine. It is sympathomimetic with multiple pharmacological effects, including analgesia and dysphoria. Ketamine binds to the allosteric phencyclidine-binding site that is located within the channel pore of the NMDA receptor and blocks the ion flux through the receptor pore; ketamine blocks the receptor noncompetitively [104,105]. The commercially available form of ketamine is a racemic mixture of S–(+) and R–(–) ketamine (also known as RS-ketamine). Upon intravenous administration, ketamine is quickly metabolized to norketamine and nordehydroketamine, which appear in the venous blood within 10 and 30 min. (R)–(–) ketamine has an approximately a four-fold smaller affinity for NMDA receptors than (S)–(+) ketamine, and it shows smaller negative psychotomimetic side effects when compared with (S)–(+) ketamine [106]. Ketamine’s pharmacological targets are not limited to NMDARs, as it may interact with different receptors and ion channels, including cholinergic, serotonin, dopamine, sigma, and opioid receptors, as well as hyperpolarization-activated cyclic nucleotide gated channels [107]. At µ, κ, and δ opioid receptors (S)–(+) ketamine is two–three times more potent than (R)–(–) ketamine [108]. The pharmacokinetic profiles of (S)–(+) and (R)–(–) ketamine are similar [106]; however, the antidepressant effect of S–(+) ketamine seems to be less potent and less lasting compared to R–(–) ketamine [109]. Since the first clinical studies demonstrated that a single sub-anesthetic dose of ketamine evokes rapid (within hours) and lasting (typically for a week) antidepressant effects, ketamine aroused substantial interest in psychiatry [110]. The usual dose in psychiatric studies is 0.5 mg/kg for 40 min [106] that results in a maximal plasma ketamine concentration of ~185 ng/mL or ~0.78 µM [111]. Evidence for the antidepressant effect achieved at doses as low as 0.1 mg/kg (five–minute intravenous infusion or intramuscular injection), resulting in a maximal plasma ketamine concentration of ~75 ng/mL (estimated ~0.32 µM), were also reported in a small crossover study in patients suffering from treatment-resistant depression [112]. (S)–(+) ketamine is effective as an antidepressant if administered via intravenous or intranasal routes [113,114,115]. Intranasal administration of (S)–(+) ketamine rapidly decreases suicidal ideation in patients suffering from depression [113]. If in humans, as is the case in mouse models of depression, (R)–(–) ketamine will prove superior potency in comparison to (S)–(+) ketamine, this may have advantages considering its fewer side effects.

The antidepressant effect of ketamine [116,117] is seemingly in agreement with the glutamatergic hypothesis of depression [118], postulating that NMDAR antagonism increases the synthesis of the brain-derived neurotrophic factor (BDNF) [119,120]. Sub-anesthetic doses of ketamine thus produce rapid antidepressant effects by transiently increasing glutamate cycling in the medial prefrontal cortex of rodents [121,122]. Within minutes, a single sub-anesthetic dose of ketamine infusion also produces a rapid and transient increase in glutamate/glutamine in the medial prefrontal cortex of patients with MDD [123]. Surprisingly, a muscarinic receptor M1 antagonist, scopolamine, and an NR_2B_-selective NMDAR antagonist, Ro 25–6981, applied at doses that produce rapid antidepressant effects in rodents, transiently increased the glutamate cycling that precedes the antidepressant effects, suggesting that a transient increase in glutamate cycling may be critical for inducing rapid antidepressant effects [122]. The speed of ketamine antidepressant effect is in striking contrast to classic antidepressants (i.e., selective serotonin reuptake inhibitors (SSRIs)) that target the monoamine system and require weeks to produce the therapeutic effect. Unlike ketamine, other NMDA receptor antagonists do not exhibit antidepressant capabilities [117,124], indicating that ketamine affects additional targets in addition to the neuronal NMDAR. Moreover, as the antidepressant effect of ketamine outlasts the lifetime of the drug in the organism [105], the continuous antagonism of NMDA receptors can also not explain the prolonged antidepressant effect. Thus, alternative mechanisms have to be considered, and mechanisms affecting synaptic connectivity [125] may well account for the prolonged antidepressant effect. As reported, ketamine increases synaptogenesis, expression of the α-amino-3-hydroxy-5-methyl-4-isoxazolepropionic acid (AMPA) receptor, dendritic spine density, and arborization of astrocytes [120,126,127]. Synaptic transmission between neurons is tightly regulated by astrocytes that release ATP, and control membrane transport of glucose and glutamate, glial-to-neuron gap junction communication, cell volume and blood flow [1], indicating their clinical relevance. In a rat chronic unpredictable stress model of depression and anxiety, the expression of glutamate transporter GLT–1 increased in a BDNF–TrkB–dependent manner 24 h after the intraperitoneal administration of a single sub-anesthetic dose of ketamine [128], indicating that GLT–1 is a potential downstream target of BDNF–TrkB signaling, which mediates the antidepressant effect. In contrast, as determined by forced swimming and novelty suppressed feeding tests, the pharmacological inhibition of GLT–1 function by dihydrokainic acid (a selective inhibitor of GLT–1 present in astrocytes) in the infralimbic cortex enhanced local glutamatergic neurotransmission and evoked rapid antidepressant response in rats [129]. Whilst the antidepressant effect mediated by the altered GLT–1 function requires further experimental clarification, multiple lines of evidences indicate that ketamine affects various aspects of astrocyte physiology [130,131,132,133,134].

## 5. Ketamine Increases cAMP Activity and Alters Vesicle–Plasmalemma Interaction in Astrocytes

**Figure 1:**
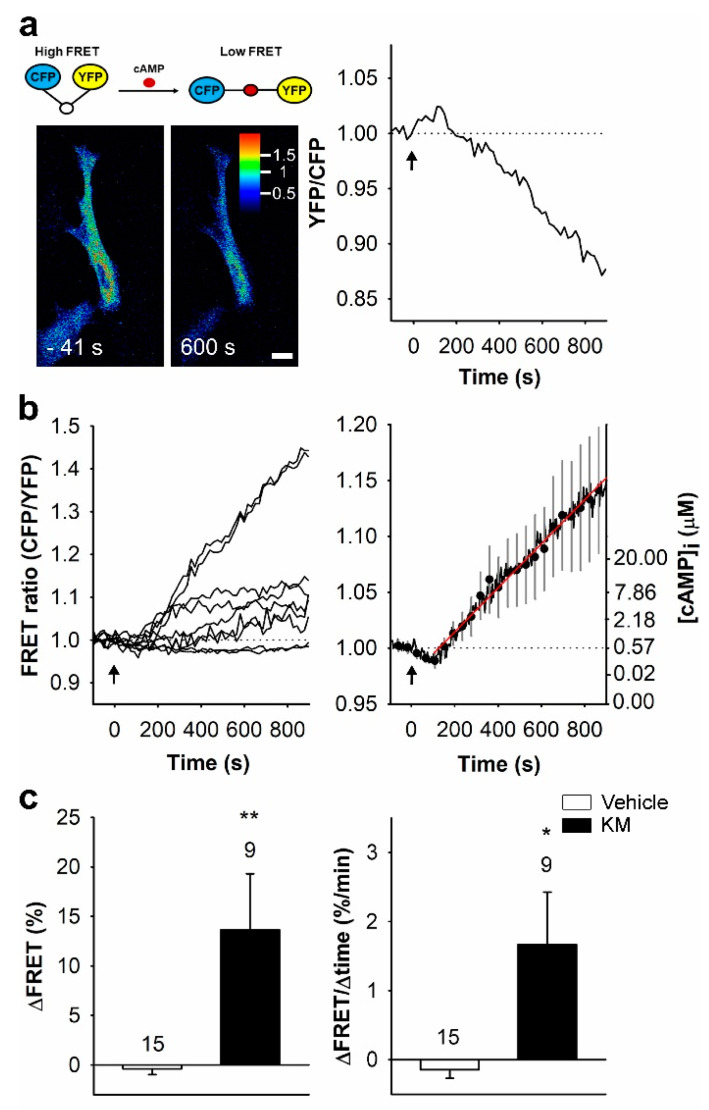
Ketamine increases [cAMP]_i_ in cultured rat astrocytes. (**a**) Schematic representation of FRET-based Epac1-camps nanosensor function (top) and pseudo-colored FRET images of an astrocyte expressing the FRET-based nanosensor Epac1-camps before (−41 s) and after (+600 s) application of 25 µM ketamine (bottom), and the corresponding normalized time resolved trace of the Epac1-camps FRET signal (YFP/CFP; right). The pseudo-colored scale indicates the YFP/CFP ratio values. Scale bar, 20 µm. (**b**) Time-resolved (left panel) and the mean time-resolved (±SEM; right panel) traces of the Epac1-camps FRET signal in astrocytes (*n* = 9) stimulated with ketamine at *t* = 0 s (black arrows). Data are expressed as the inverted FRET signal (CFP/YFP). A ketamine-evoked increase in the FRET signal reflects an increase in [cAMP]_i_. The initial rate of change in [cAMP]_i_ (red line; right panel) was determined by fitting the regression line to the FRET signal increase (*k* = 2.2 ± 0.6%/min). The ordinate on the right displays the values of [cAMP]_i_ estimated from the equation: [cAMP]_i_ = EC_50_ × ((R − R_min_)/(R_max_ − R))^1/n^ [148]. (**c**) Mean amplitude (±SEM; ΔFRET; left panel) and initial rate of the FRET signal change (ΔFRET/Δtime; right panel) in controls (white bars) and in astrocytes treated with ketamine (KM, black bars). Changes in the FRET signal are expressed as percentages of the initial values. Numbers above the error bars report number of cells analyzed. Mann–Whitney U test: * *p* < 0.05, ** *p* < 0.01. Reproduced with permission from Lasic and colleagues [149].

In protoplasmic astrocytes, cAMP plays a role in the establishment of an arborized structure [135,136,137] that covers a territorial domain [32], within which a single rodent astrocyte may contact up to 100,000 synapses through fine, sub–micrometer sized processes [16,138,139]. These fine processes, in addition to astrocyte-derived factors, influence synaptic activity [140,141,142,143,144] and promote synaptogenesis [145]. In cultured rat astrocytes, ketamine induces a delayed and lasting increase in intracellular cAMP concentration ([cAMP]_i_) (Figure 1) [130,134] that attenuates the vesicular delivery of channels to the plasmalemma [146], and may modify vesicle cargo release or uptake of the extracellular molecules through the altered structure of the fusion pore [130,132]. By affecting the translocation of G_αs_ from lipid rafts to non-raft membrane microdomains and thus allowing increased functional coupling of G_αs_ and adenylyl cyclase, ketamine facilitates cAMP signaling in a NMDA receptor independent manner in C6 glioma cells. Importantly, in depressed patients, reduced cAMP signaling was reported, whereas treatment with SSRI increased cAMP levels [147].

Ketamine also increases phosphorylation of the cAMP response element-binding protein (CREB), which in turn, increases the expression of BDNF [134] playing a prominent role in the pathophysiology of MDD [150,151,152]. Beside neurons, astrocytes also synthesize BDNF, and are targeted by antidepressants [150,153,154]. In primary cortical astrocyte culture, SSRI antidepressants fluoxetine and paroxetine and the tricyclic antidepressants imipramine and amitriptyline upregulate levels of BDNF mRNA in a monoamine-independent manner [154,155,156,157,158], possibly through a direct effect on the extracellular signal regulated kinase- and p38 mitogen activated protein kinase-dependent signaling pathway. At the single vesicle level, ketamine suppresses the stimulus-evoked exocytotic release of BDNF [132] and attenuates ATP-evoked increases in [Ca^2+^]_i_ in cultured cortical astrocytes isolated from rat [132] and neocortical astrocytes in mice [133]. Reduced BDNF release from single vesicles and diminished frequency of full exocytotic events [132] can be explain by attenuated stimulus-evoked calcium signaling in ketamine-treated astrocytes. By modifying Ca^2+^ entry through TRP channels that contribute to the replenishment of the endoplasmic reticulum store (capacitive function) [159] and to the plateau phase of the stimulus-evoked Ca^2+^ transient [160], ketamine may diminish stimulus-evoked Ca^2+^ signaling [161,162]. Ketamine also inhibits glutamate transmission from astrocytes to neurons and disrupts synchronization of astrocytic slow inward currents, presumably mediated by the extrasynaptic GluN_1_/GluN_2B_ receptors [163].

Ketamine applied at clinically relevant concentrations modulates the interaction of astrocytic vesicles with the plasmalemma by stabilizing a narrow fusion pore [130]. Following treatment with sub-anesthetic concentrations of 0.25 and 2.5 µM ketamine [164], astrocyte vesicles lapse into a state of repetitive fusion pore opening and closing [130] that may hinder cargo discharge [165] or uptake and vesicle membrane recycling that affects the retention of receptors, transporters and ion channels on the astrocyte surface [49]. The molecular mechanisms underlying flickering pore activity are still debated. At physiological pH, ketamine permeates membranes [166] and accumulates inside acidified vesicles as a protonated weak base [167]. As a charged molecular entity, it may electrostatically alter the anisotropicity of the membranous pore [168] and directly affect the pore [169]. By inhibiting vesicle endocytosis [130], ketamine could obstruct BDNF uptake into astrocytes [170,171] and favor increased extracellular levels of BDNF that are more likely to signal to nearby synapses and support long-term potentiation via sustained TrkB activation, leading to the enhancement of synaptic strength between neurons [150,151,152].

## 6. Ketamine Attenuates Mobility of K_ir_4.1 Vesicles and Reduces Surface Density of K_ir_4.1

Two studies in animal models of depression [172,173] revealed a distinctive burst firing activity in neurons of lateral habenula (LHb) that was causally linked to reduced extracellular concentration of K^+^ ([K^+^]_o_) [172]. Regulation of [K^+^]_o_ is essential for optimal neuronal function and astrocytes utilize an inward rectifying K^+^ channel (K_ir_4.1) that acts as a major conduit for the movement of K^+^ between extracellular space and astrocytes [174,175]. In rat model of depression, astrocytic K_ir_4.1 was found upregulated at the transcript, protein and functional levels [172]. Over-expressed K_ir_4.1 hyperpolarizes membrane potential and causes burst firing of LHb neurons, which results in a depression-like phenotype that is mimicked by lowering [K^+^]_o_. Conversely, pharmacological blockade or disruption of K_ir_4.1 function depolarizes membrane potential and causes tonic firing of LHb neurons. As overexpressed astrocyte K_ir_4.1 lowers [K^+^]_o_ and favors burst firing and depression [173], astrocytes play a role in regulating the brain neuronal circuits involved in mood and motivation. Ketamine infusion into LHb blocks neuronal burst firing and causes rapid antidepressant effects [172,173]. Whether ketamine also inhibits K_ir_4.1 is currently unknown. However, antidepressants of the tricyclic and SSRI class, such as nortriptyline and fluoxetine reversibly inhibit K_ir_4.1 activity [176,177,178]. Ketamine, on the other hand, reduces cytoplasmic mobility of K_ir_4.1 vesicles [146], likely by affecting motor protein-driven directional mobility of vesicles along microtubules [179]. As proposed by Bensel and colleagues [180], ketamine, in addition to propofol, binds to kinesins and/or the kinesin-β-tubulin interface and diminishes kinesins’ ability to transport cargo. By binding to druggable allosteric binding sites that form transiently when kinesin motor domain binds to the microtubule lattice, ketamine reduces kinesin microtubule affinity and promotes motor protein detachment from the microtubule, which results in reduced run length (total distance kinesin travels per microtubule encounter) [180]. Over time, attenuated kinesin-driven vesicle mobility may result in diminished K_ir_4.1 density at the astrocyte surface [146], which heightens extracellular K^+^ concentration, alleviating depressive symptoms in animal models of depression [172], and possibly also in depressed humans [110]. Pharmacological modulation of K_ir_4.1 activity in the brain circuits involved in regulation of mood and motivation might be a way to treat MDD.

## 7. Ketamine Increases Cholesterol Content in the Exofacial Leaflet of the Astrocyte Plasmalemma

Flawless operation of the central nervous system relies on synaptic transmission between neurons [47,181,182]. Synaptic connectivity and synaptic function is regulated by perisynaptic astrocytes [1,62]. As earlier in vitro studies revealed, cultured retinal ganglion neurons by themselves form only few synapses that are functionally immature [47,181,182]. Upon the addition of astrocytes, the total number of synapses on neurons increases by sevenfold [182]. Moreover, astrocytes are also required to stabilize these synapses, because synapses formed in their presence are quickly lost when astrocytes are removed [47]. One of the key synapse-promoting signals is cholesterol [183]. In the brain, cholesterol is derived almost entirely from in situ synthesis by brain cells [184]. In neuronal ganglion culture, the number of synapses, and spontaneous and evoked synaptic activity increases within three days after the addition of glial-conditioned medium or cholesterol (5 µg/mL) (Goritz et al., 2005). As the appearance of most synapses in the developing brain temporally and spatially coincides with the development of astrocytes, synapse formation very likely depends on astrocyte-derived cholesterol [47]. Although neurons produce sufficient cholesterol to survive and grow, they require astrocyte-derived cholesterol to form abundant new synapses and to mature these synapses (Mauch et al., 2001).

**Figure 2:**
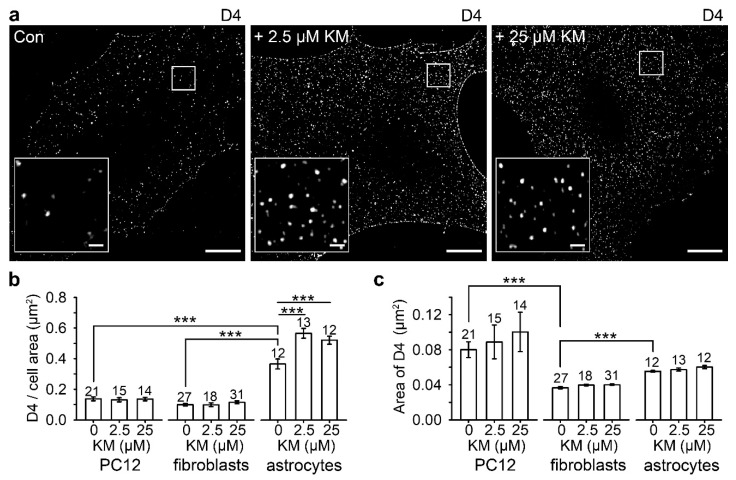
Ketamine (KM) increases the surface density of cholesterol-rich plasmalemmal domains in astrocytes. (**a**) Structured illumination microscopy images displaying mCherry-D4-labeling in non-treated controls (Con) and ketamine-treated astrocytes (2.5 µM and 25 µM KM, respectively). Insets show mCherry-D4-labeled domains at a higher magnification. Scale bar (inset), 10 µm (1 µm). (**b**) The density of the membrane cholesterol domains (the number of cholesterol domains (D4) normalized to the cell surface area) significantly increased in astrocytes after treatment with 2.5 µM (0.57 ± 0.03 D4/µm^2^) and 25 µM KM (0.52 ± 0.03 D4/µm^2^), when compared with controls (0.37 ± 0.03 D4/µm^2^) (*** *p* < 0.001, Holm–Sidak one-way ANOVA). Ketamine did not affect the density of the cholesterol-rich domains in the PC12 cells and fibroblasts. The density of D4-positive domains was higher in astrocytes (0.37 ± 0.03 D4/µm^2^) when compared with PC12 cells (0.14 ± 0.01 D4/µm^2^) and fibroblasts (0.10 ± 0.01 D4/µm^2^) (*** *p* < 0.001, Kruskal–Wallis test). (**c**) The average area of cholesterol-rich domains in vehicle-treated controls and in ketamine-treated cells (2.5 µM and 25 µM) did not differ in the PC12 cells, fibroblasts and astrocytes, but it differed between different cell types. The area of D4-positive domains was significantly higher in PC12 cells (0.080 ± 0.009 µm^2^) and astrocytes (0.056 ± 0.001 µm^2^) than in fibroblasts (0.037 ± 0.001 µm^2^) (*** *p* < 0.001, Kruskal–Wallis test). The data in the graphs are reported as mean ± SEM. Numbers above the bars represent the number of cells analyzed. Modified and reproduced with permission from Lasic and colleagues [149].

Suspicious loss of astrocyte number in brain regions important for mood, motivation and cognition reported in post-mortem studies of patients suffering from MDD, schizophrenia and bipolar disorder [64,65,66,67,68,69] may also indicate insufficient supply of astrocyte–derived cholesterol to neurons that require cholesterol to build up the enormous membrane surfaces of their dendrites, axons and synapses [185,186], and to maintain functional synapses [144]. In hippocampal neurons [187], spinal cord [188] and retina [47,181,183], soluble and contact-dependent astrocyte signals stimulate the formation of synapses, whereas ketamine instigates rapid redistribution of cholesterol in the astrocyte plasmalemma [149]. Cholesterol-enriched lipid rafts labeled with a fluorescent cholesterol-specific membrane binding domain D4 of perfringolysin O [189,190] already redistribute within 30 min after ketamine is added to astrocytes (Figure 2).

Increased cholesterol content in the exofacial leaflet of astrocyte plasmalemma may transiently boost cholesterol flux towards neurons where cholesterol may counteract the reduction in dendritic spine number and function of neurons observed in an animal model of depression [191] or reduction in the number of synapses observed in MDD subjects [192]. The mechanism by which ketamine increases the surface density of plasmalemmal cholesterol in astrocytes is currently unclear. Overall increase in astrocyte cholesterol production is unlikely, since no increases in serum level of cholesterol were observed after ketamine administration (120 and 140 mg/kg) to male Wistar rats, nor did intraperitoneal administration (1 mg/kg) for 6 days affect the synthesis of cholesterol [193,194]. As an alternative, a vesicle-based mechanism may account for increased surface density of cholesterol in astrocyte plasmalemma. Sub-anesthetic ketamine doses evoke prolonged flickering activity of narrow fusion pore that hinders endocytosis [130], which may contribute to a slow increase in cholesterol content in the plasmalemma, as cholesterol-enriched membrane domains are not effectively internalized into cells via endocytosis. Enriched cholesterol content in the exofacial leaflet of the astrocyte plasmalemma [149] may favor flux of cholesterol to neurons, where it is required for changes in synaptic plasticity [185] and strengthening of excitatory synapses necessary for the improvement of depressive behavior [195]. This proposal, however, requires further experimentation.

## 8. Conclusions

**Figure 3:**
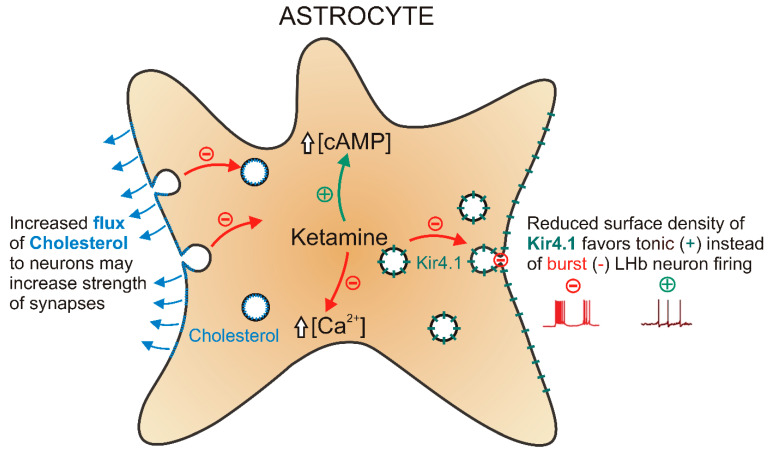
Ketamine-evoked changes in astrocyte plasticity. Ketamine inhibits (-) stimulus-evoked calcium signaling in astrocytes and enhances (+) cAMP production. Ketamine suppresses exocytosis of gliosignaling molecules and vesicular delivery of K_ir_4.1 (green) to the plasmalemma. Ketamine elevates cholesterol (blue) content in the exofacial leaflet of the astrocyte plasmalemma. Lateral habenula (LHb). Not drawn to scale.

Numerous studies in animal models of depression and post-mortem samples from patients with major depressive disorder [196] revealed a decrease in the density or number of astrocytes. Astrocyte atrophy suggests a causative role for astrocytes in ethiopathogenesis of MDD. As ketamine affects diverse astrocyte functions (Figure 3) within the similar time domain as the clinically relevant antidepressant effect setting in [110,117,197], it is possible that ketamine partially exerts its antidepressant effect via astrocytes that modulate neural excitability and synaptic transmission.

By attenuating mobility of vesicular K_ir_4.1, ketamine reduces the surface density of K_ir_4.1 [146] that regulates extracellular K^+^ and tunes the pattern of action potential firing in LHb neurons in a rat model of depression [172,173]. Ketamine further increases [cAMP]_i_ [149], which plays a role in the establishment of ramified cell structure [135,136,137], enabling an astrocyte to contact up to 100,000 synapses and promote their formation [145]. Ketamine also instigates rapid redistribution of cholesterol in the astrocyte plasmalemma [149], which may further boost cholesterol flux to neurons [185], leading to sustained strengthening of excitatory synapses necessary for improvement of depressive behavior [195]. Thus diverse, but not mutually exclusive mechanisms may act synergistically to evoke changes in synaptic plasticity leading to sustained strengthening of excitatory synapses necessary for the antidepressant effect of ketamine.
